# Epigenetic regulation of somatostatin and somatostatin receptors in neuroendocrine tumors and other types of cancer

**DOI:** 10.1007/s11154-020-09607-z

**Published:** 2020-10-21

**Authors:** M.J. Klomp, S.U. Dalm, M. de Jong, R.A. Feelders, J. Hofland, L.J. Hofland

**Affiliations:** 1 Department of Internal Medicine, Division of Endocrinology, Erasmus MC, Rotterdam, The Netherlands; 2 Department of Radiology & Nuclear Medicine, Erasmus MC, Rotterdam, The Netherlands

**Keywords:** Neuroendocrine tumors, Cancer, Somatostatin, Somatostatin receptor, Epigenetic regulation

## Abstract

Both somatostatin (SST) and somatostatin receptors (SSTRs) are proteins with important functions in both physiological tissue and in tumors, particularly in neuroendocrine tumors (NETs). NETs are frequently characterized by high SSTRs expression levels. SST analogues (SSAs) that bind and activate SSTR have anti-proliferative and anti-secretory activity, thereby reducing both the growth as well as the hormonal symptoms of NETs. Moreover, the high expression levels of SSTR type-2 (SSTR2) in NETs is a powerful target for therapy with radiolabeled SSAs. Due to the important role of both SST and SSTRs, it is of great importance to elucidate the mechanisms involved in regulating their expression in NETs, as well as in other types of tumors. The field of epigenetics recently gained interest in NET research, highlighting the importance of this process in regulating the expression of gene and protein expression. In this review we will discuss the role of the epigenetic machinery in controlling the expression of both SSTRs and the neuropeptide SST. Particular attention will be given to the epigenetic regulation of these proteins in NETs, whereas the involvement of the epigenetic machinery in other types of cancer will be discussed as well. In addition, we will discuss the possibility to target enzymes involved in the epigenetic machinery to modify the expression of the SST-system, thereby possibly improving therapeutic options.

## Introduction

Somatostatin receptors (SSTRs) are a family of G protein coupled receptors, of which different subtypes exist, i.e. SSTR1, SSTR2, SSTR3, SSTR4 and SSTR5. Alternative splicing of *SSTR2* RNA generates two splice variants: SSTR2a and SSTR2b which differ in length. SSTRs can be activated by the neuropeptide somatostatin (SST), of which two isoforms are known, i.e. somatostatin-14 (SST-14) and somatostatin-28 (SST-28), both having high affinity for SSTRs [1, 2]. SST is produced by different organs in both the central nervous system, e.g. hypothalamus, and in other organs including pancreas, stomach and intestine. It is synthesized in response to multiple biological signals, for instance neurotransmitters, hormones and neuropeptides [3]. SSTR-expressing cells are found abundantly in human tissues, such as the brain, pituitary and the gastrointestinal tract [4]. The SST-system is therefore involved in regulating multiple physiological processes. This is mediated via several pathways that are activated upon binding of SST to SSTRs, which results in either inhibition of hormone secretion and cell proliferation, or induction of apoptosis [5, 6].

**Fig. 1:**
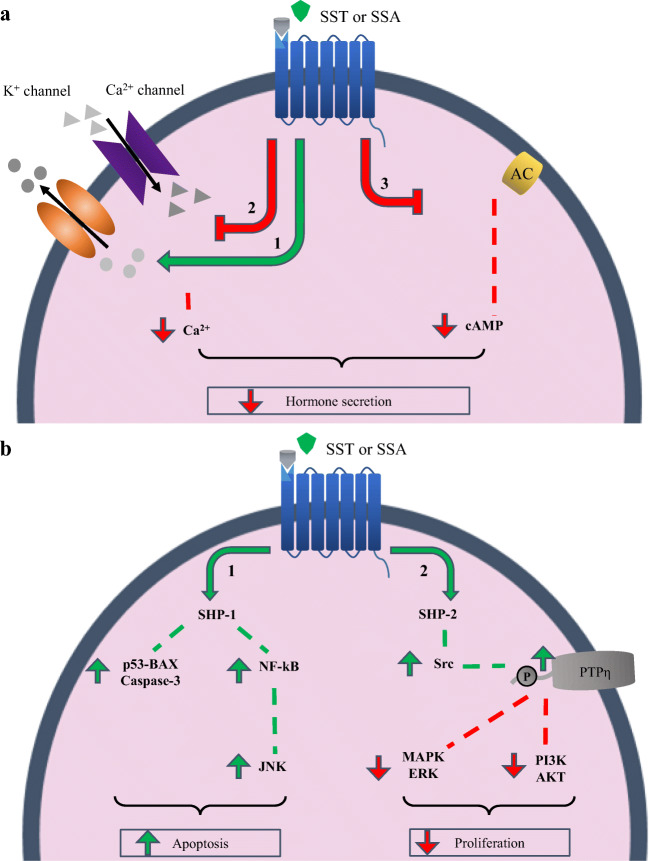
(a) Upon binding of SST or SSAs, (1,2) K^+^ channels are activated and Ca^2+^ channels are inhibited, resulting in decreased Ca^2+^ levels, and (3) adenylyl cyclase (AC) activity is inhibited thereby reducing intracellular cAMP levels. This results in inhibition of hormone secretion. (b) Activation of the SST-system also results in anti-tumoral activity: (1) SHP-1 is activated, thereby increasing pro-apoptotic and reducing anti-apoptotic proteins, and (2) SHP-2 is activated which results in activation of PTPη by Src-mediated phosphorylation. PTPη causes inhibition of pathways physiologically involved in cell proliferation. In both figure a and b, effects induced by SSTR activation are indicated by green (enhanced) or red (reduced) arrows

SSTR-mediated anti-secretory effects are induced via two main pathways: (1) inhibition of adenylyl cyclase (AC) resulting in reduced levels of cyclic AMP (cAMP) and (2) activation of K^+^-channels and inhibition of voltage-dependent Ca^2+^-channels resulting in reduced intracellular Ca^2+^ levels (Fig. 1a). Upon binding of SST to SSTR, src-homology phosphatase (SHP) proteins are activated which are involved in regulating cell proliferation and apoptosis. SHP type-1 (SHP-1) is involved in inducing apoptosis by increasing pro-apoptotic proteins such as the p53-Bax-caspase-3 pathway and by increasing JNK expression resulting in inhibition of anti-apoptotic proteins (Fig. 1b). SHP type-2 (SHP-2) activation results in Src activity, which phosphorylates PTPη. As a result, MAPK/ERK and PI3K/AKT proteins will be inactivated, causing upregulation of proteins involved in inhibiting proliferation (Fig. 1b).

In addition to its pivotal role in physiological processes, the SST-system also plays an important role in neuroendocrine tumors (NETs). SSTR2 is highly expressed on NETs, thereby being an important target for therapy [7, 8]. Moreover, aberrant SSTR expression has also been reported for other cancers, including breast cancer [9], colorectal cancer [10], prostate cancer [11, 12] and larynx cancer [13]. For prostate cancer, it was demonstrated that SSTR2 and SSTR5 are mostly expressed, i.e. in 34.8% and 56.5%, respectively [12]. In contrast, SSTR1 was expressed abundantly in 90% of primary breast cancer tissues, whereas SSTR2 and SSTR5 were expressed in a lower number of cases, i.e. in 34.4% and 44.4% of the examined cases, respectively [14]. In this review, we aim to highlight the role of epigenetic mechanisms involved in the regulation of the SST-system, consisting of both SSTRs and the neuropeptide SST. We will focus on the regulation of both proteins in different types of tumors, with particular emphasis on NETs. Moreover, we will discuss the possibility to target the epigenetic machinery in order to modulate either SSTR or SST expression. An improved understanding of the epigenetic regulation involved in the SST-system may result in new approaches to either improve the efficacy of current treatments or expand therapeutic options for patients with tumors expression low SSTR levels.

## Somatostatin and somatostatin receptors in NETs

NETs arise from neuroendocrine cells found throughout the entire body. Consequently, NETs are a heterogeneous disease that can develop at different locations, such as the gastrointestinal tract, pancreas and lung, accounting for 54%, 22% and 12% of the NETs, respectively [15]. NETs are divided in functional and non-functional tumors. Functional NETs are frequently characterized by an overproduction of hormones, such as serotonin, gastrin or insulin. As the tumor is frequently already metastasized upon diagnosis [15], treatment options are limited. Currently, resection is still the only curative therapy option [16], and is only possible for the minority of NET patients due to the presence of metastases.

In the past decades, several treatment options were developed that improved patient outcome [17], of which some target the SSTR2. In literature, a high expression of SSTR2 has been described in NETs. For example, in a study performed by Mizutani et al. [18], SSTR mRNA levels were measured for 13 NET samples derived from several locations. It was shown that SSTR2 is expressed in all cases. In another study, examining 112 small intestine NETs (siNETs), 19 pancreatic NETs (pNETs) and 42 NETs derived from other locations, it was demonstrated that 65%, 76%, 90%, 86% and 93% of all cases were recognized by the expression of SSTR1, SSTR2, SSTR3, SSTR4 and SSTR5, respectively [7]. Upon discriminating low and high expression levels, SSTR2 was expressed most frequently, i.e. 51% of the examined NETs were recognized by high SSTR2 levels. Especially, pNETs and siNETs were most frequently characterized by the expression of SSTR2. These high SSTR2 expression levels paved the way for SSTR2-targeted treatments in NETs, including unlabeled somatostatin analogues (SSAs) [19] and peptide receptor radionuclide therapy (PRRT) [20, 21]. SSAs have potent anti-secretory effects and thereby reduce symptoms related to the overproduction of bioactive substances by tumors in a significant proportion of NET patients [22, 23]. In addition, various studies have demonstrated that SSAs have tumor growth inhibitory actions in NET patients. Octreotide long-acting release (LAR) and lanreotide autogel are both SSAs with high affinity for SSTR2 [24]. Patients with well-differentiated metastatic midgut NETs benefited from octreotide LAR treatment, as demonstrated in a placebo-controlled study with 85 patients enrolled. This study showed a significantly increased time to progression from 6 to 14.3 months between the control and octreotide LAR treatment groups, respectively [25]. Similar, a phase III study with lanreotide autogel in metastatic enteropancreatic NET patients reported a significantly increased progression-free survival compared to placebo [26].

The generation of radiolabeled SSAs further improved treatment options for patients with SSTR-positive tumors**.** PRRT with radiolabeled SSAs induced significant anti-tumor effects in NET patients with metastatic SSTR-expressing bronchial and gastroenteropancreatic NETs, in terms of response rates, progression-free survival, overall survival and safety profile [27, 28]. In the study by Brabander et al. [27], the objective tumor response rate was 39%, achieving 2% complete response and 37% partial response. Median overall survival rates depend on the primary location of the tumor, i.e. 52, 60 and 71 months for bronchial, midgut and pancreatic NETs, respectively. In the NETTER-I phase III clinical trial, it was demonstrated that 4 cycles of PRRT with [^177^Lu]Lu-[DOTA,Tyr^3^]-octreotide ([^177^Lu]Lu-DOTA-TATE) in combination with octreotide LAR resulted in longer progression-free survival and higher response rates in patients with advanced midgut NETs, compared to high-dose octreotide LAR treatment alone [29]. Together, these results led to the FDA and EMA approval of [^177^Lu]Lu-DOTA-TATE for SSTR-positive gastroenteropancreatic NETs. Additionally, studies demonstrated a possible role of PRRT as neo-adjuvant treatment option for non-functioning pNETs [30], emphasizing the broad scope of PRRT. Although treatment with SSAs and PRRT have both clearly proven their value for the treatment of NETs, complete responses are still rare [25, 31], leaving room for therapy improvement. Moreover, not all NET patients are eligible for SSTR-targeted treatments due to variable SSTR2 expression levels among patients [32, 33]. NETs display a low frequency of mutations and chromosomal aberrations [34], and no mutations in *SSTR2* have been identified thus far. This suggests other mechanism(s) underlying these heterogeneous SSTR expression levels, e.g. epigenetic regulation.

### Epigenetic regulation

**Fig. 2:**
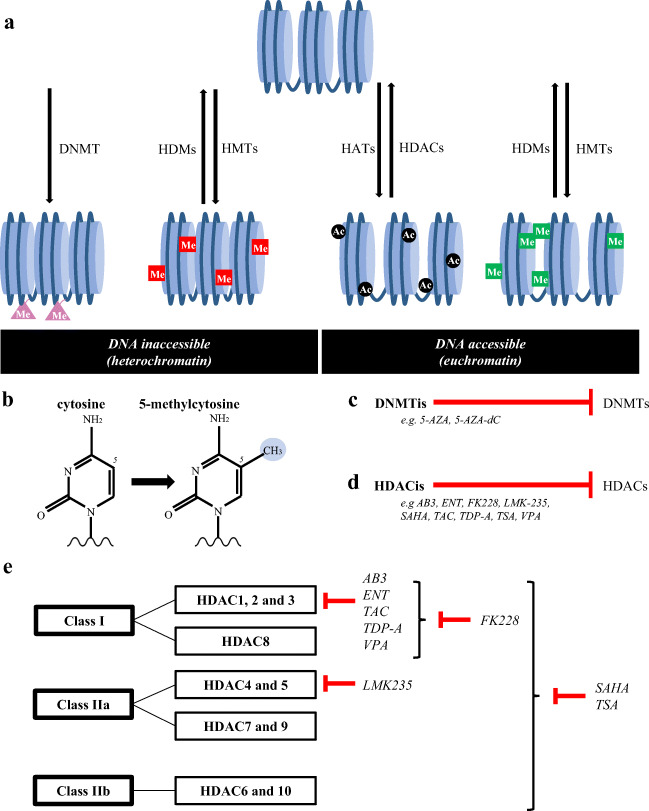
(a) Epigenetic modifications can modify both DNA and histones. DNA methylation and inactivating histone methylation stimulate heterochromatin, resulting in inaccessible DNA and therefore no gene transcription. Histone acetylation and activating histone methylation stimulate euchromatin, thereby stimulating gene transcription. Histone methylation can therefore both be inactivating and activating, depending on which lysine residue is modified on which histone. Examples of inactivating histone methylation marks (indicated in red) are H3K9me2/3, H3K27me2/3 and H4K20me3, and activating histone methylation marks (indicated in green) are H3K4me2/3, H2K36me3 and H3K79me3. All epigenetic modifications are catalyzed by enzymes: (1) DNA methylation by DNMTs, (2) histone methylation marks by HMTs and HDMs, and (3) histone acetylation marks by HATs and HDACs. (b) DNMTs are involved in DNA methylation in which cytosine residues are converted into 5-methylcytosine residues. (c,d) Epigenetic drugs have been developed inhibiting certain groups of enzymes involved in epigenetic modifications, i.e. DNMTis and HDACis targeting DNMTs and HDACs, respectively, both stimulating transcriptionally active euchromatin. (e) HDACis often target multiple HDACs within HDAC class I, IIa and/or IIb. AB3, entinostat (ENT), tacedinaline (TAC), thailandepsin-A (TDP-A) and valproic acid (VPA) target HDAC1, 2 and 3; romidepsin (FK228) targets all HDAC protein within class I; LMK235 targets HDAC4 and 5 within class IIa; vorinostat (SAHA) and trichostatin A (TSA) target HDAC proteins within class I, IIa and IIb. [46, 104–106]

Cell-specific gene transcription is regulated by epigenetic modifications which are heritable during cell divisions. However, as the DNA sequence itself is not changed, these modifications are also characterized by their reversibility [35, 36]. Over the last years, epigenetic modifications have become an important field of interest, demonstrating their pivotal role in physiological processes, such as their role in cell differentiation and development [37]. Moreover, thorough investigations have demonstrated that the epigenetic machinery is also involved in the development of diseases, e.g. neurodevelopmental disorders and autoimmune diseases, as well as the development of cancer [38–41]. For its role in the development of cancer, the epigenetic machinery is involved in the activation of proto-oncogenes and/or inactivation of tumor suppressor genes, such as *RB*, *P16* and *BRCA1* [41]. Epigenetic modifications can regulate gene expression at different levels. In this review, we will specifically focus on modifications targeting the DNA and the histones (Fig. 2a).

One of the major epigenetic mechanisms are histone modifications. Specific histone-modifying enzymes can stimulate the formation of either condensed, inactive heterochromatin or decondensed, active euchromatin (Fig. 2a). Acetylation and methylation of amino acids are the most frequently observed modifications at the N-terminal tails of the histones. Acetylation on lysine amino acids leads to a reduction of positive charges on the surface of histones, resulting in loss of interactions between DNA and histones. This in turn results in euchromatin formation which stimulates gene transcription. Histone acetyltransferases (HATs) and histone deacetylases (HDACs) are responsible for the addition and removal of acetyl groups on lysine residues, respectively. Whereas histone acetylation is linked to transcriptional activation, histone methylation can either be repressive or activating. This depends on which lysine residue (K) on which histone (H3, H4) is modified, and the extent of methylation, i.e. di- or tri-methylation (me2, me3). H3K9me2/3, H3K27me2/3 and H4K20me3 are inhibiting histone methylation marks, and H3K4me2/3, H2K36me3 and H3K79me3 are known as important activating histone marks. The process of histone methylation is mediated by histone lysine methyltransferases (HMTs) and histone demethylases (HDMs) [42, 43].

Another relevant epigenetic modification targets cytosine residues in the DNA. Methyl groups are transferred to the fifth carbon of the cytosine nucleobases by DNA methyltransferases (DNMTs) (Fig. 2a, b). This process is mainly catalyzed by three subtypes, i.e. DNMT1, DNMT3a and DNMT3b. DNMT1, interacting with ubiquitin-like with PHD and RING finger domain (UHRF) proteins, is involved in maintaining methylation profiles during replication. DNMT3a and DNMT3b are both involved in de novo transfer of methyl groups. The catalytic activity of DNMT3a and DNMT3b is increased upon association with DNMT3L, which does not have catalytic activity on its own. DNA methylation often occurs on cytosine residues followed by guanine residues or in CpG islands which are frequently present within the promotor region. In response to DNA methylation, transcription factors are no longer able to bind. Moreover, specific inhibitory proteins bind to the DNA upon methylation resulting in repression of transcription, such as methyl-CpG-binding domain (MBD) proteins and zinc-finger proteins. [44, 45].

Of note, there is a strong interplay between histone modifications and DNA methylation. For example, activating histone modifications prevent binding of DNMTs, thereby enhancing gene transcription. Moreover, DNMTs can interact with HMTs and HDAC, together stimulating the silenced heterochromatin state. Additionally, repressing MBD proteins interacting with methylated DNA are involved in regulating histone modifications, leading to transcriptional repression [44].

Based on the growing knowledge about the epigenetic machinery and its key role in gene transcription, drugs have been developed that target the enzymes involved in the above-mentioned processes. DNA methyltransferase inhibitors (DNMTis, Fig. 2c) and histone deacetylase inhibitors (HDACis, Fig. 2d) both stimulate gene transcription, as these epigenetic drugs inhibit DNMTs and HDACs, respectively. There are several HDAC subtypes, leading to the development of subtype-specific HDACis. In short, mainly based on both the homology with yeast HDACs and their function, human HDACs are divided in four classes; class I, II, III and IV, of which HDACs class II is subdivided in class IIa and IIb. The HDACis discussed in this review are targeting one or multiple classes, constituted of several HDAC proteins: epigenetic drugs targeting class I (HDAC1, 2, 3 and 8), class IIa (HDAC4, 5, 7 and 9) and/or class IIb (HDAC6 and 10) (Fig. 2e) [46, 47].

**Fig. 3:**
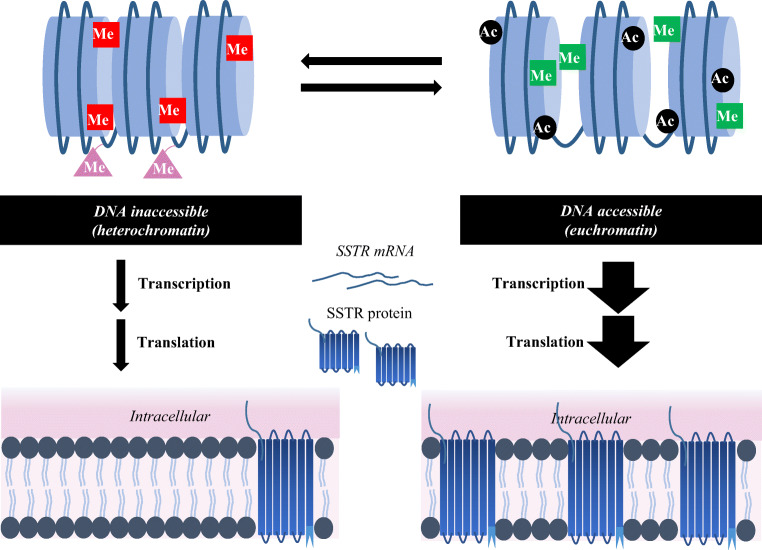
Activating histone marks (indicated in green) stimulate euchromatin, resulting in more gene transcription. Epigenetic drugs may be used to stimulate euchromatin, in order to increase the expression of certain proteins. Thereby, it may be possible to increase the expression of targets for therapy, e.g. SSTR2 in NET patients with insufficient expression levels for treatment

Summarizing, DNMTis and HDACis can both be used to specifically target and change the epigenetic machinery and profile. These epigenetic drugs can therefore create many possibilities to modify gene and thus protein expression levels, e.g. SSTR expression, in order to expand current therapy options for cancer (Fig. 3).

### Epigenetic regulation in NETs

The important role of epigenetics in NET tissue has already been demonstrated in several clinical studies. In a cohort of pNET patients, significant upregulation of multiple HDAC subtypes was reported, including HDAC3, nuclear HDAC4, nuclear and cytoplasmic HDAC5, cytoplasmic HDAC8, HDAC9, nuclear HDAC10 and HDAC11. More specifically, HDAC1, HDAC2, nuclear HDAC5 and HDAC11 were significantly elevated in high grade (G3) pNETs, compared to low-grade pNETs (G1 and G2). Further analysis showed that especially upregulation of nuclear HDAC5 was significantly associated with a reduced disease-free survival and overall survival [48]. Additionally, the HDACi entinostat (ENT) reduced the activity of proteins involved in the progression from primary to metastatic disease [49]. This suggests that histone acetylation marks could be a target for therapy. In line with changes in histone acetylation patterns, aberrant DNA methylation patterns have been described in NET tissue, emphasizing the important role of epigenetics in regulating gene expression involved in numerous processes such as cell cycle, cell death, cell growth and DNA repair [34, 40]. In an extensive review by Mafficini et al. [50], the genetic and epigenetic alterations in pNETs and siNETs have been described. In this overview, promotor hypermethylation of multiple genes was described, for instance hypermethylation of RASSF1A and CDKN2A, both being tumor suppressor genes involved in regulating cell cycle arrest, apoptosis and/or senescence. Additionally, inactivating mutations in HMTs were described, further suggesting deregulation of the epigenetic machinery.

Altogether, there is strong evidence that the epigenetic machinery is highly involved in the pathophysiology of NETs. It might even be speculated that this system is also involved in the regulation of SST and SSTR expression and signaling. Elucidating the exact regulation of SSTR in NETs is of high importance, as this protein has an important function as target for therapy.

## Epigenetic regulation of SSTR

### Epigenetic regulation of SSTR in NETs

As mentioned above, SSAs are a cornerstone medical treatment modality for NETs, targeting SSTR2 which is often expressed at a high level in NETs. The genomic DNA of human *SSTR2* contains multiple transcription start sites (TSSs). Two TSSs are located 82–93 nucleotides upstream [51, 52] from the translation start codon with an initiation element *inr* in close proximity. This *inr* is involved in regulating gene transcription in the absence of a TATA-box as transcription factors are able to bind to the E-box present within this *inr* [53]. Another TSS is located further upstream and contains a CpG island [54]. As CpGs are often the target for epigenetic modifications, it is likely that epigenetic regulation is involved in controlling *SSTR2* gene expression via this TSS. This suggests that deregulation of the epigenetic machinery may also influence tumoral SSTR2 expression. To elucidate the role of epigenetic regulation in NET patients, different NET cell lines have been used. These include cell lines derived from pNETs (i.e. BON-1 and QGP-1), pulmonary NETs (i.e. NCI-H727), siNETs (i.e. GOT-1) and medullary thyroid cancer (i.e. TT and MZ-CRC-1), which are all characterized by their own basal SSTR2 expression levels.

In pNET cells lines BON-1 and QGP-1, both DNA methylation and histone modifications regulate SSTR2 expression. In comparison with other NET cell lines, BON-1 and QGP-1 cells are both characterized by relatively low SSTR2 expression levels. However, *SSTR2* mRNA levels are still relatively high compared to cell lines derived from other types of cancer [54]. QGP-1 cells demonstrated low *SSTR2* promotor methylation rates at only 2% in the 8 CpG islands examined [55]. Similar observations were made for the pancreatic BON-1 cells, characterized by slightly higher SSTR2 expression levels compared to QGP-1 cells. Low (~3%), or even unmeasurable levels of DNA methylation were found in the genomic region surrounding the TSS in BON-1 cells [54, 55]. The low levels of DNA methylation and relatively low SSTR2 expression levels could be related to the involvement of DNA methylation in other regions, as the above described studies only focus on specific areas in the promotor region. Torrisani et al. [54] showed an inverse association between the level of CpG island methylation and *SSTR2* mRNA levels within several cell lines, including the pNET cell line BON-1. Additionally, transfection of a methylated *SSTR2* promotor in BON-1 cells induced silencing of the *SSTR2* promotor. This effect was caused by the absence of binding of transcription factor specificity protein-1, a protein involved in regulating the basal *SSTR2* promotor activity [54]. Together, these observations support the potential of *SSTR2* promotor methylation to suppress SSTR2 expression. Moreover, acetylation on histone 3 was present in both BON-1 and QGP-1 cells [56]. The involvement of histone acetylation was further confirmed by Veenstra et al. [55]. In conclusion, both DNA methylation and histone acetylation are likely involved in regulating SSTR2 expression, i.e. triggering heterochromatin and euchromatin, respectively.

The above-mentioned associations between epigenetic markers and SSTR2 expression levels suggest that epigenetic drugs could potentially stimulate SSTR expression in NET cells. The use of epigenetic drugs, such as DNMTis and HDACis, may stimulate euchromatin, thereby promoting *SSTR2* gene transcription. This approach can especially be important for NET patients not eligible for SSTR2-mediated therapies due to insufficient or undetectable SSTR expression levels.

#### Modulation of SSTR expression in vitro in NET cell lines

Successful stimulation of SSTR2 through attenuation of methylation has been demonstrated in BON-1 cells by treatment with DNMTis 5-aza-2′-deoxycytidine (5-AZA-dC) or 5-azacitidine (5-AZA), as shown by significantly enhanced uptake of [^68^Ga]Ga-DOTA-TOC [57]. Further analysis of 5-AZA-dC pretreatment demonstrated increased *SSTR2* mRNA and SSTR2 protein expression levels, which increased over time. Moreover, the uptake of [^68^Ga]Ga-DOTA-TOC was clearly enhanced at human 5-AZA-dC therapeutic serum concentrations, whereas effects were barely observed at lower concentrations. Based on these data, a time- and dose-dependency was suggested. The efficacy of 5-AZA-dC in modulating SSTR2 expression is investigated in several other studies. A seven-day treatment schedule resulted in enhanced *SSTR2* mRNA expression levels in both BON-1 and QGP-1 cells using 100 nM and 50 nM, respectively. Receptor functionality was subsequently demonstrated with internalization studies using [^125^I]I-[Tyr^3^]octreotide, reporting a significantly increased 1.85-fold uptake in BON-1 cells [55]. In line with this, significantly increased SSTR2 protein levels in BON-1 cells after a 3 day exposure to 2.5 μM 5-AZA-dC were also observed in the study by Jin et al. [58]. However, in another study, it was demonstrated that a 3 day exposure to a lower dose of 5-AZA-dC (2 μM) had no significant effects on *SSTR2* mRNA expression levels in BON-1 cells [54]. As the experimental set-up is similar in terms of cell line and exposure time, the results support data the above mentioned of Taelman et al. [57] of a dose-dependent response. As both time- and dose-dependency are clearly suggested, a precise treatment regimen may be important parameter for study outcome.

In addition to DNA methylation, histone acetylation is also likely involved in regulating SSTR2. HDACis therefore also gained great interest as a novel therapeutic strategy to stimulate SSTR2 expression. Several HDACis have been tested in QGP-1 cells, which has led to contradictory results. Whereas Veenstra et al. [55] demonstrated increased internalization of radiolabeled SSAs after valproic acid (VPA) treatment, *SSTR2* mRNA levels were significantly decreased by 1 mM VPA. This indicated other modes of action, e.g. fast redirection of the receptor to the membrane after internalization, via yet unknown epigenetic mechanisms. Contrary to these findings, significantly increased *SSTR2* mRNA levels were reported after VPA treatment by Guenter et al. [59] when using a higher VPA dosage (4 mM). Other HDACis, such as romidepsin (FK228), vorinostat (SAHA) and AB3, provided similar results as significant upregulation was demonstrated on *SSTR2* mRNA expression level. Unfortunately, western blot analysis could not confirm SSTR2 upregulation in QGP-1 cells upon HDACi treatment [59]. In contrast to this, the use of the HDACi LMK235 provided more convincing results, as this treatment resulted in increased SSTR2 protein expression levels [56]. An epigenetic mechanism of action was confirmed by an augmented acetylation of histone 3 upon HDACi-treatment. Of note, LMK235 has high affinity for HDAC4 and HDAC5, both belonging to HDAC class IIa, whereas all the other tested HDACis either target multiple HDAC-classes or specifically target HDAC class I. This may suggest that HDAC4 and HDAC5 are highly involved in inducing euchromatin, thereby enabling *SSTR2* transcription in QGP-1 cells. The effects of HDACi-treatment in BON-1 cells were more consistent than the results in the QGP-1 cell line. A screen of several HDACis (i.e. scriptaid, dacinostat, panobinostat, trichostatin A (TSA), SAHA, phenylbutyrate, FK228 and tacedinaline (TAC)) demonstrated enhanced uptake of radiolabeled SSAs by BON-1 cells, reaching statistical significance for most HDACis [57]. Further analysis of cells treated with TAC demonstrated significantly increased *SSTR2* mRNA and SSTR2 protein expression levels. In line with these results, significantly increased *SSTR2* mRNA levels were described after TSA treatment by Torrisani et al. [54]. Furthermore, protein expression levels were significantly increased upon TAC treatment [58], specifically inhibiting HDAC1–3, further supporting the enhanced uptake described by Taelman et al. [57]. FK228, SAHA and AB3 were also able to enhance *SSTR2* mRNA significantly within 24 h, and even demonstrated increased protein expression levels after 48 h treatment [59]. Moreover, it was shown that upon LMK235 treatment the level of acetylation on histone 3 was increased, providing a dose-dependent increase of SSTR2 protein after a one day treatment [56].

Furthermore, the effects of VPA in BON-1 cells were evaluated in various studies. VPA treatment resulted in an increased level of acetylation on histone 4, thereby confirming changes in the epigenetic machinery. In line with this, SSTR2b protein expression was increased [60]. This VPA-augmented *SSTR2* expression level was confirmed at mRNA level upon short- (24–28 h) and long-term (7 days) VPA treatment [55, 59, 61]. Furthermore, a 7.2-fold stimulated SSTR2 protein expression level was observed [59], while the functionality of increased SSTR2 expression was further confirmed by a significantly increased uptake of radiolabeled SSAs [55, 57]. Receptor functionality was also confirmed by an increased efficacy of camptothecin-somatostatin conjugates after VPA treatment, as demonstrated by reduced BON-1 cell proliferation [60]. These results suggest that SSTR2 expression is more easily modified in BON-1 cells than in QGP-1 cells, and the effects seem to be less dependent on the targeted HDAC classes.

In addition to pNETs, the effect of HDACis was examined in both small intestinal (i.e. GOT-1 and KRJ-I) and pulmonary (i.e. NCI-H727) NETs, characterized by variable SSTR2 expression levels. Expression levels in GOT-1 cells exceed that of NCI-H727 cells, which are both characterized by higher expression levels compared to BON-1 and KRJ-I cells [58, 62]. Although there has been some debate about the origin of KRJ-I cells [63, 64], VPA treatment increased *SSTR2* mRNA levels in these cells [61]. Studies with the small intestinal cell line GOT-1 are still limited. The effect of HDACi treatment was solely examined upon monotherapy with either VPA [61] or TAC [58], resulting in a statistically significant ~2-fold increased SSTR2 protein expression level after TAC treatment, while no significant changes in mRNA expression were observed after VPA treatment. For comparison, a similar treatment schedule did change *SSTR2* significantly in BON-1 and KRJ-I cells.

In the pulmonary NET cell line NCI-H727, SSTR2 protein expression level was not changed significantly upon TAC treatment [58]. However, in another study, HDACis thailandepsin-A (TDP-A), FK228, SAHA, VPA and AB3 were able to increase *SSTR2* mRNA levels significantly when high dosages were used [65]. Western blot analysis confirmed over 2.5-fold upregulation in all conditions. Further examination of TDP-A-treated NCI-H727 cells demonstrated both receptor functionality and increased receptor-mediated uptake of [^68^Ga]Ga-DOTA-TATE. Equal concentrations of TDP-A, FK228, SAHA, VPA, and AB3 showed SSTR2 protein upregulation in the TT medullary thyroid cancer cell line up to 3-fold, whereas these effects were not observed in the MZ-CRC-1 medullary thyroid cancer cell line. Of note, MZ-CRC-1 cells are characterized by high basal SSTR2 expression level compared to TT cells.

Studies have also focused on combining epigenetic treatments, e.g. the combination of DNMTis and HDACis. In BON-1 cells, the combination treatment of 5-AZA-dC and VPA had additive or synergetic effects as demonstrated by higher *SSTR2* mRNA levels and higher uptake of [^125^I]I-[Tyr^3^]octreotide compared to either monotherapy [55]. Moreover, this combination of epigenetic drugs also significantly increased [^125^I]I-[Tyr^3^]octreotide uptake in QGP-1 cells, while effects of both monotherapies didn’t result in significant changes. In addition, combination of 5-AZA-dC and TAC gave synergistic effects as well, in terms of [^68^Ga]Ga-DOTA-TOC uptake and cell survival [57]. A similar combination treatment of 5-AZA-dC and TAC was examined in the BON-1, NCI-H727 and GOT-1 cell line by another research group, demonstrating significantly increased SSTR2 protein expression levels of 8.31-, 1.56- and 2.06-fold, respectively [58]. Additive effects were demonstrated for BON-1 cells, whereas this was not evidently observed for H727 and GOT-1. Thus, the effects in H727 cells and GOT-1 cells were less pronounced compared to results obtained with BON-1 cells.

Altogether these studies clearly suggest the involvement of the epigenetic machinery in the regulation of SSTR2 expression in NET cells. Although convincing results in QGP-1 were only induced upon LMK235 treatment, results obtained with other cell lines suggest that especially NET cell lines with low (i.e. BON-1) or intermediate (i.e. NCI-H727 and TT cells) SSTR2 expression levels are susceptible to epigenetic drug treatment, whereas upregulation in NET cell lines with high SSTR2 expression levels is more limited (i.e. GOT-1 and MZ-CRC-1 cells). This supports the concept of epigenetic therapy for NET patients with insufficient SSTR2 expression, thereby potentially making more patients eligible for treatment with (radiolabeled) SSAs. The studies described above are summarized in Table 1. Of note, DNMTis (i.e. 5-AZA-dC) and some of the HDACis (i.e. VPA, TAC, SAHA, FK228) have been tested in clinical trials. Based on published pharmacokinetic parameters, it can be concluded that the drug concentrations used in the studies described above are within the same order of magnitude or even within the achievable human therapeutic range.

**Table 1:** Overview of in vitro studies with their main findings relevant for this review, focusing on modifying SSTR expression in NET cell lines using DNMTis or HDACis

Cell types	Epidrug	Treatment regimen	Main findings	Ref
BON-1	Screen of several DNMTis and HDACis, e.g. 5-AZA-dC and TAC	75 ng/mL 5-AZA-dC or 500 ng/mL TAC; time-dependency experiment (1–3 days)	- TAC and 5-AZA-dC increased the uptake of [^68^Ga]Ga-DOTA-TOC most efficiently; *SSTR2* mRNA and SSTR2 protein expression levels were also significantly increased - Observed effects are time- and dose-dependent - Synergetic effects upon combination therapy in terms of [^68^Ga]Ga-DOTA-TOC uptake and cell survival	[57]
BON-1 QGP-1	5-AZA-dC, VPA	BON-1: 100 nM 5-AZA-dC and/or 2.5 mM VPA; 7 days QGP-1: 50 nM 5-AZA-dC and/or 1 mM VPA; 7 days	- Low *SSTR2* CpG island methylation around transcription start site; ~3% in BON-1, ~2% in QGP-1 - All treatments increased *SSTR2* mRNA levels and uptake of [^125^I]I-[Tyr^3^]octreotide significantly in BON-1; enhanced effects for combination therapy *- SSTR2* mRNA levels and uptake of [^125^I]I-[Tyr^3^]octreotide increased after combination therapy in QGP-1 - Treatment of QGP-1 with VPA decreased *SSTR2* mRNA levels and enhanced uptake of [^125^I]I-[Tyr^3^]octreotide non-significantly, suggesting other mechanisms of action - Histone acetylation more likely involved in regulating SSTR2 expression than histone methylation	[55]
BON-1 NCI-H727 QGP-1 GOT-1	5-AZA-dC, TAC	2.5 μM or 5.0 μM 5-AZA-dC and/or 2.5 μM or 5.0 μM TAC; 3 days	- SSTR2 protein expression levels in QGP-1 undetectable before and after HDACi treatment - Combination treatment induced statistically significant upregulation of SSTR2 protein expression in BON-1, GOT-1 and NCI-H727; maximum increase of 8.31-fold in BON-1 - TAC significantly enhanced SSTR2 expression in BON-1 and GOT-1; 5-AZA-dC in BON-1 and NCI-H727	[58]
BON-1	5-AZA-dC, TSA	2 μM 5-AZA-dC and/or 150 nM TSA; 3 days	*- SSTR2* upstream promotor not methylated - Significantly upregulated *SSTR2* mRNA expression levels upon TSA and combination therapy - Statistically significant correlation between *SSTR2* mRNA expression and CpG island methylation in upstream promotor	[54]
BON-1 QGP-1	TDP-A, SAHA, VPA, FK228, AB3	2 nM or 6 nM TDP-1, 1 μM or 3 μM SAHA, 1 mM or 4 mM VPA, 2 nM or 6 nM FK228 or 1 μM or 3 μM AB-3 1 day for RT-qPCR, 2 days for further analysis	*- SSTR2* mRNA levels significantly increased after 1 day treatment with 3 μM SAHA, 4 mM VPA, 6 nM FK228 and 3 μM AB3, in both BON-1 and QGP-1 - SSTR2 protein levels not evidently increased in QGP-1; maximum increase of 1.7-fold - SSTR2 protein levels clearly enhanced in BON-1; maximum increase of 7.2-fold - Increased functional SSTR2 density on cell surface for 6 nM FK228 in BON-1	[59]
BON-1 QGP-1	LMK235	0.08 μM, 0.31 μM, 1.25 μM, 5.0 μM and 20 μM; 1 or 2 days	- Dose-dependent increased acetylation on histone 3 upon LMK235 treatment - Dose-dependent increased SSTR2 protein level in BON-1 - SSTR2 protein levels detectable in QGP-1 after high concentration LMK235 treatment	[56]
BON-1	VPA	2 mM or 4 mM; time-dependency experiment (3, 6, 18, 36 and 72 h)	- Time-dependent increased level of acetylation on histone 4 - Reduced activity of HDAC4 after chronic treatment - Increased SSTR2b and decreased SSTR1, SSTR3, SSTR4 and SSTR5 protein expression levels - VPA enhanced anti-proliferating effects of camptothecin-somatostatin conjugates	[60]
BON-1 KRJ-I GOT-1	VPA	4 mM; 28 h	- Significantly increased *SSTR*2 mRNA expression level in BON-1 and KRJ-I	[61]
NCI-H727, MZ-CRC-1 TT	TDP-A, SAHA, VPA, FK228, AB3	2 nM or 6 nM TDP-1, 1 μM or 3 μM SAHA, 1 mM or 4 mM VPA, 2 nM or 6 nM FK228 or 1 μM or 3 μM AB-3 1 day for RT-qPCR, 2 days for further analysis	*- SSTR2* mRNA levels significantly increased in NCI-H727 after highest-dose HDACi treatment; VPA and TDP-A also increased expression at lower dose - SSTR2 protein levels evidently increased; minimum increase of 2.5-fold in NCI-H727 - TDP-A treatment significantly increased uptake of [^68^Ga]Ga-DOTA-TATE in NCI-H727 - SSTR2 protein upregulated in TT after HDACi treatment; limited effects in MZ-CRC-1 which are characterized by higher basal SSTR2 expression levels compared to TT	[65]

#### Modulation of SSTR expression in vivo in NET xenograft-models

Based on the in vitro results discussed above, the effects of epigenetic drugs were also tested in vivo using NET-bearing mice. Direct anti-proliferative effects can be induced by HDACi treatment. Reduced xenograft growth was observed after AB3, VPA, TDP-A, FK229 and ENT treatment in BON-1, GOT-1, TT and/or H-STS NET tumor-bearing mice, although statistical significance was not reached in all studies [49, 60, 61, 66–68]. The combination treatment of VPA and camptothecin-somatostatin conjugate significantly reduced BON-1 tumor growth by 66%, compared to 17% and 42% for both monotherapies, respectively [60]. Tumors were not resected in this study and HDACi-upregulated SSTR2 expression was therefore not confirmed. Encapsulation of the HDACi TDP-A in micelles functionalized with either KE108 [66] or octreotide [68] reduced tumor volume with 92% and 74%, respectively. In both these studies, significant differences were found between the effects observed after treatment with TDP-A-loaded targeted micelles compared to TDP-A-loaded non-targeted micelles. Similar results were described for AB3-encapsulted KE108-functionalized micelles tested in medullary thyroid cancer TT xenografts [67]. According to the authors, the enhanced effects for TDP-A-loaded targeted micelles can be explained by the combination of both passive and active tumor targeting ability, i.e. enhanced permeation retention effect and efficient targeting of SSTRs, respectively. Unfortunately, tumors were not further analyzed to confirm changes in SSTR2 expression levels upon HDACi treatment. Although these data are not available, it may also be hypothesized that the enhanced effects upon treatment with TDP-A-loaded or AB3-loaded targeted micelles are caused by the fact that the HDACis are targeted to the SSTR2-expressing tumor cells, resulting in enhanced receptor expression due to HDACi-mediated changes in the epigenetic machinery. This SSTR2 upregulation may lead to increased therapeutic efficacy as more functionalized micelles will be targeted to the tumor cells. However, this hypothesis requires further investigations.

In the study published by Taelman et al. [57], it was demonstrated that 5-AZA-dC significantly increased the uptake of [^68^Ga]Ga-DOTA-TOC in BON-1 tumor-bearing mice in a dose-dependent manner, resulting in increased tumor-to-background and tumor-to-kidney ratios. Moreover, a blocking study demonstrated SSTR-specific uptake after HDACi treatment, indicating SSTR-upregulation. As a result, tumors could be visualized using PET/CT-imaging modality. In addition to this study using a DNMTi, two studies have been published in which SSTR2 expression levels were examined by PET/CT-scans upon inhibition of HDAC class I proteins. For BON-1 tumor-bearing mice, significantly increased standard uptake values (SUVs) were observed on a PET/CT-scan after [^68^Ga]Ga-DOTA-TATE injection when mice were pre-treated with FK228 [59]. A similar effect was observed in mice with NCI-H727 xenografts that were treated with TDP-A. This study showed a trend towards SSTR upregulation following HDACi-treatment, although statistical significance was not reached due to differences in individual tumor size and uptake of [^68^Ga]Ga-DOTA-TATE [65].

### Epigenetic regulation of SSTR in other cancer types

Studies focusing on other types of cancer showed that deregulation of SSTR is also often established by epigenetic mechanisms. For colorectal cancer (CRC), the *SSTR2* promotor was characterized by enhanced methylation levels, which was associated with reduced SSTR2 expression [69]. Similar results were obtained in head and neck squamous cell carcinomas (HNSCC). Here, higher methylation levels of SSTR1 were detected compared to adjacent normal mucosal tissue, which correlated with several clinicopathologic features [70]. This was confirmed in squamous cell carcinoma cell lines, exhibiting low *SSTR1* mRNA levels and high levels of promotor methylation in comparison to normal cell lines. In line with these results, increased methylation levels on CpG sites present within the *SSTR2* promotor region were also described for laryngeal squamous cell carcinomas [71]. Moreover, the *SSTR1* promotor was frequently methylated in Epstein-Barr virus (EBV) positive primary gastric cancer samples (67%), whereas this was not the case in EBV-negative primary gastric cancer samples [72]. In line with this result, *SSTR2, SSTR3* and *SSTR5* mRNA expression levels were also reported to be reduced in gastric cancer samples compared to paired normal gastric tissue [73]. For SSTR2, this was confirmed by Kim et al. [74] by a negative correlation between *SSTR2* methylation and gene expression in human gastric tumor tissue. In general, SSTR expression is reduced in cancer because of methylation of the promotor region. This suggests the involvement of the epigenetic machinery in deregulated SSTR expression in cancer. Epigenetic drugs can therefore potentially modulate SSTR expression and thus therapeutic opportunities.

#### Modulation of SSTR expression in vitro and in vivo in other types of cancer

The involvement of the epigenetic machinery in other cancer types is further supported by studies aiming to increase SSTR expression using epigenetic drugs. The effect of VPA was evaluated in several human cell lines, i.e. hepatocellular carcinoma cells [75], small cell lung cancer cells [76] and cervical cancer cells [77, 78]. The epigenetic mechanism of action of VPA was confirmed by western blot analysis in hepatocellular carcinoma and lung cancer cells, as demonstrated by a decreased expression of HDAC4 protein and increased acetylation on histone 4. Likely as a result of this altered acetylation pattern, *SSTR2* was upregulated, i.e. a 20.6 and 7.4-fold increase, respectively. Therapeutic efficacy was increased in small cell lung cancer cells when VPA treatment was combined with camptothecin- or colchicine-somatostatin conjugates as shown by decreased cell growth in vitro. For cervical cancer cells, expression of *SSTR* subtypes were also changed upon VPA treatment. Here, VPA increased expression of *SSTR2*, *SSTR3* and *SSTR5* in a dose-dependent manner, while *SSTR1* expression levels were downregulated. The VPA-induced SSTR2 upregulation, resulted in enhanced effects of cytotoxic-somatostatin conjugates, both in vitro and in vivo [77, 78].

The epigenetic machinery was also shown to be involved in the regulation of SSTR2 expression in pancreatic cancer cell lines. Upon epigenetic drug treatment with DNMTi 5-AZA-dC or HDACi TSA, *SSTR2* mRNA levels were increased, with even stronger upregulation observed for the combination treatment. These results suggest the involvement of both DNA methylation and histone acetylation [54]. The possibility to modulate *SSTR2* transcription by DNA methylation was confirmed by Gailhouste et al. [79], as *SSTR2* mRNA levels were upregulated after 5-AZA treatment, resulting in reduced cell growth upon treatment with SSAs. Upregulation of *SSTR4* and *SSTR5* was also reported in response to DNMTi treatment, thereby emphasizing the important role of DNA methylation in controlling the expression of several receptors within the SST-pathway.

*SSTR5* expression in primary human laryngeal squamous cell carcinoma tissue demonstrated to be significantly lower compared to corresponding normal tissue. Low expression levels were confirmed in cell lines. Further analysis of these cancer cell lines demonstrated that methylation of exon 1 of the *SSTR5* gene is likely involved in downregulation of the protein. The involvement of histone modifications was also confirmed, as treatment with 5-AZA and/or TSA upregulated *SSTR5* mRNA expression levels. For the AMC-HN-8 cell line, the presence of active and inactive histone modifications in the *SSTR5* promotor were examined. Activating histone mark H3K4me3 was enriched upon 5-AZA-dC or combination treatment, activating histone mark H3K9ac was enriched upon TSA or combination treatment, and repressive histone mark H3K9me2 was decreased upon 5-AZA-dC or combination treatment. In this extensive study by Wang et al. [80], the involvement of both DNA methylation and histone modifications was therefore clearly demonstrated in the regulation of SSTR5 in laryngeal squamous cell carcinomas cell lines.

Moreover, 5-AZA-dC and TSA treatment increased *SSTR5* mRNA expression levels in a castration-resistant prostate cancer cell line [81]. Combining DNMTi and HDACi treatment had additive effects in this cell line in terms of *SSTR5* expression, whereas such effects were not observed in androgen-sensitive cell lines, suggesting cell type-specific responses. This cell type-specific response was further confirmed as 5-AZA-dC treatment was associated with *SSTR1* hypomethylation in androgen receptor-positive prostate cancer cell line, whereas this was not observed in an androgen-receptor negative prostate cancer cell line [82].

Data has also suggested the involvement of epigenetic mechanisms in the controlling SSTR2 expression in gastric cancer. In line with this, 5-AZA-dC and/or TSA treatment restored both *SSTR2* and *SSTR4* mRNA expression in 75% of the examined gastric cell lines, with the greatest effects observed upon combination therapy [74]. Upon comparing EBV-positive AGS gastric cancer cells and EBV-negative AGS gastric cancer cells, it was demonstrated that EBV-positive AGS cells are characterized by enhanced activity of DNMT3b and higher SSTR1 CpG island methylation levels. Further analysis showed that the viral latent membrane protein 2A (LMP2A), expressed upon EBV infection, is involved in DNMT3b upregulation. Treatment of EBV-positive AGS cells with 5-AZA-dC resulted in increased *SSTR1* mRNA levels. Of note, this was not observed in the EBV-negative gastric cell line. The latter cell line is characterized by very low *SSTR1* CpG island methylation levels compared to EBV-positive AGS cells [72, 83]. This suggests that DNMTis are only efficient in enhancing SSTR levels in cells characterized by high DNA methylation levels.

Summarizing, these data demonstrated that, in line with the epigenetic regulation involved in SSTR2 expression in NETs, the epigenetic machinery plays an important role in the regulation of multiple SSTRs in other cancer types as well. Moreover, it is possible to increase SSTR expression in a number of cancer types by epigenetic drug treatment.

## Epigenetic regulation of SST in cancer

As discussed above, activation of SSTR by SST can induce several effects, e.g. inhibiting cell proliferation and hormone secretion, and promoting apoptosis. SST is therefore known has a protein with anti-proliferative and anti-secretory activity. This was further supported in a recently published paper, showing that knock out of SST in the BGC823 gastric cancer cell line resulted in an increased capacity for migration and invasion in vitro [84]. Due to its role, SST expression in cancer is evaluated extensively in order to find new biomarkers or to expand current therapeutic options. For NET patients, long-acting SSAs increase progression-free survival [25, 26], confirming the anti-proliferative activity of SST upon SSTR activation. In addition, preclinical studies showed that the SST*-*SSTR interaction has tumor suppressor activity in certain tumors [85]. This raises the question whether deregulation of SST expression in NETs has impact of NET function as well. However, to the best of our knowledge, no reports have been published yet about the epigenetic regulation of SST in NETs.

On the other hand, the regulation of SST expression in gastric cancer has been subject to research. SST knock-down experiments in the GES-1 cell line resulted in a lowering of cells in the G0/G1 phase, suggesting an important role of SST in cell proliferation [86]. Moreover, a high DNA methylation level of the SST promotor and its assocation with undetectable SST expression has been described in seven gastric cancer cell lines [87]. Li. et al. [88] confirmed *SST* promotor hypermethylation in gastric cancer tissue. However, the authors were unable to validate a reduction in *SST* mRNA expression in human tissue using 10 pairs of tumor and adjacent non-tumorous tissue (*p* = 0.074). Contradictory to this, reduced *SST* mRNA and SST protein expression levels have been described in gastric carcinoma samples throughout multiple studies using larger cohorts of patients. Reduced *SST* mRNA and SST protein expression levels thereby both correlated with increased *SST* DNA methylation levels [73, 87, 89].

For renal cell carcinomas, published results are equivocal. Ricketts et al. [90] demonstrated hypermethylation both in cell lines and in primary tissue samples. In these tissue samples, it was shown that tumor-specific hypermethylation of the *SST* promotor was associated with reduced *SST* mRNA expression levels. Contradictory, Morris et al. [91] reported promotor hypermethylation only in renal cancer cell lines, whereas this was not observed in any of the analyzed primary renal cell carcinoma tissue samples.

The involvement of the epigenetic machinery in the regulation of SST has also been demonstrated in several studies focusing on colon cancer and CRC. CpG sites within the *SST* gene were hypermethylated in different stages of tumorous samples, i.e. 94%, 100%, 94% and 57% for adenomas with low-grade dysplasia, adenomas with high-grade dysplasia, CRC and metastatic-CRC, respectively [92]. Likely as a result of this observed hypermethylation in CRC, the *SST* mRNA level was decreased in CRC compared to normal tissue, as demonstrated with microarray data [93]. In a small pilot study with only 4 samples collected from patients with pre-neoplastic colorectal sessile serrated adenomas, *SST* hypermethylation was demonstrated in all examined patients [94]. These results indicate that, among others, the downregulation of SST may be involved in the development of CRC. In line with this, *SST* promotor methylation is increased in CRC, associating with reduced expression levels [69, 93, 95]. In agreement with the results observed in CRC samples, *SST* promotor methylation levels were also significantly increased when focused specifically on primary colon cancer samples compared to normal colonic mucosae, i.e. 88% versus 47%, respectively [96].

Limited information is available about the epigenetic regulation of SST in other types of cancer. *SST* promotor hypermethylation was shown in pancreatic PANC-1 cells. Here, CpG methylation rates of 96–98% were associated with extremely low *SST* mRNA levels [79]. Moreover, knockdown of DNMT1 increased *SST* expression, emphasizing the role of DNA methylation and thus the epigenetic machinery in the regulation of SST expression. Furthermore, analysis of glioblastoma multiforme tissue samples demonstrated both *SST* hypermethylation on CpG sites and a 80.5-fold downregulated *SST* expression level compared to control brain tissue [97]. *SST* hypermethylation has also been reported for human tissue derived from cervical cancer [98] and anal cancer [99], and both in cell lines and human tissue derived from esophageal carcinomas [100], gliomas [101] as well as HNSCC [70, 102, 103]. For esophageal carcinomas, gliomas and HNSCC, a negative correlation with mRNA expression was found. It has even been suggested that SST may be used as a methylation-based biomarker for the prognosis and/or diagnosis of HNSCC [102], CRC [69, 95], cervical [98] and anal cancer [99].

There is a possibility that the presence of endocrine cells in the examined tissues affect the outcome of these studies, for example the presence of enteroendocrine cells in control colorectal and CRC tissue. These endocrine cells are characterized by the expression of SST, and differences in the number of these cells in normal and tumor tissue, and thus the level of SST expression, may bias the conclusions focusing on downregulated SST expression levels in tumor tissue. However, *SST* hypermethylation, which is reported for several types of tumors, still suggests that SST, with its anti-proliferating and anti-secretory effects, is under the control of the epigenetic machinery. To the best of our knowledge, no data are available with respect to the role of histone acetylation within this process. In line with SSTR2 regulation, SST expression can therefore be modified by the use of epigenetic drug inhibitors targeting DNA methylation, i.e. DNMTis.

### Modulation of SST expression in vitro and in vivo

In vitro studies have demonstrated that the DNMTi 5-AZA-dC modulates SST expression. 5-AZA-dC has been shown to induce demethylation of the *SST* gene and/or concomitantly increased SST protein expression levels in cell lines derived from colon cancer [96], renal cell carcinoma [90] and esophageal cancer [100]. Similar results were observed in the PANC-1 pancreatic cancer cell line upon 5-AZA treatment [79]. Results were also confirmed in vivo. Subcutaneous inoculation of 5-AZA pre-treated cells in athymic nude mice resulted in reduced tumor growth. Moreover, 5-AZA treatment of PANC-1 xenograft-bearing mice induced a significant reduction in tumor volume. Examination of the resected tumors showed that *SST* mRNA levels were significantly increased after 5-AZA treatment. Moreover, the *SST* promotor was demethylated at CpG sites upon epigenetic drug treatment.

Additionally, in the AGS gastric cancer cell line, the effect of 5-AZA-dC was dose-dependent. A lower dose (1.6 μM; 3 days treatment) had no effect on *SST* mRNA levels in the AGS cell line [89], whereas a higher dose (5 μM; 3 days treatment) reduced *SST* DNA promotor methylation levels and restored mRNA expression to detectable levels. Combination treatment of 5-AZA-dC and TSA even further increased *SST* mRNA levels [87]. Studies with other gastric cancer cell lines showed higher *SST* mRNA levels upon 5-AZA-dC in a subset of the tested cell lines [89], suggesting cell line-specific responses. Cell line-specific responses were also reported for gliomas [101], only demonstrating *SST* upregulation upon 5-AZA-dC treatment in cell lines characterized by promotor hypermethylation. The glioma cell lines U251 and SF767 are characterized by 51.6% and 77.1% methylation of the *SST* promotor, respectively. Upon 5-AZA-dC treatment, *SST* mRNA levels were significantly increased. Of note, these effects were not observed in SF126 cells, characterized by only 14.2% methylation. This suggests that DNMTi are only effective in cell lines with high promotor methylation levels. Upon inducing SST expression, there was enrichment of activating histone marks H3Ac and H3K4me3, and reduction of inhibiting mark H2K9me3, suggesting an interplay between the promotor region and chromatin structure.

Altogether, several lines of evidence suggest that DNA methylation as well as histone modifications are involved in deregulated SST expression in various types of cancer. Moreover, *SST* expression can be modulated by the use of epigenetic drugs, thereby further supporting the involvement of the epigenetic machinery.

## Conclusion

In conclusion, understanding of the epigenetic mechanisms involved in the regulation of the expression of the SST-system is important for both NETs and other tumor types. A detailed analysis of this system potentially opens up new possibilities to develop or improve treatment options for different types of SSTR-expressing tumors, including NETs. Although studies clearly prove the involvement of epigenetics in the regulation of SSTRs and SST expression in vitro, more in-depth studies are required to confirm the ability to upregulate SSTR2 by using epigenetic drugs in vivo. Proper analysis to confirm the mechanism of action of epigenetic drugs are often lacking, e.g. examining histones profiles, immunohistochemistry, RT-qPCR and/or autoradiography to confirm increased SSTR expression. 

Moreover, receptor-specificity should be determined after epigenetic drug treatment. Since most of the knowledge on the epigenetic regulation of the SST-system is derived from in vitro studies in cell lines and experimental tumor models, future studies should also focus on the role of epigenetic marks in determining SSTR expression in primary NET tissues from patients. Moreover, the safety profile of epigenetic drugs on healthy tissue should be assessed as these drugs may potentially upregulate physiological SSTR2 expression which possibly results in enhanced (radio)toxicity in non-targeted organs. Known and future insights in the epigenetic regulation of SST and SSTR in NETs may result in the development of epidrug-based treatment modalities aiming to increase SST and SSTR2 expression. Increased intra-tumoral SST expression may in turn lead to anti-secretory and anti-proliferative effects, whereas increased SSTR2 expression could improve tumor visibility with SSTR-scintigraphy and enhance tumor response to (radiolabeled) SSAs. This may in particular be beneficial for patients with low or insufficient SSTR expression. Moreover, future studies, also including safety, are required to define the optimal dose and treatment duration for mono- and combination therapy with DNMTis and/or HDACis.
